# Benchmarking: contexts and details matter

**DOI:** 10.1186/s13059-017-1258-3

**Published:** 2017-07-05

**Authors:** Siyuan Zheng

**Affiliations:** Department of Genomic Medicine, The University of Texas MD Anderson Cancer Center, Houston, Texas 77030 USA

## Abstract

Benchmarking is an essential step in the development of computational tools. We take this opportunity to pitch in our opinions on tool benchmarking, in light of two correspondence articles published in *Genome Biology*.

Please see related Li et al. and Newman et al. correspondence articles: www.dx.doi.org/10.1186/s13059-017-1256-5 and www.dx.doi.org/10.1186/s13059-017-1257-4

Technological advances have profoundly changed cancer research over the past two decades. Increasingly affordable, it is now routine to sequence the genome and transcriptome of a sample. As a result, we are accumulating unprecedented volumes of data. For instance, the Genomic Data Commons hosted by the National Cancer Institute contains 14,551 cancer samples totaling a storage space of 5 petabytes as of May 2017 [1]. The sheer amount suggests data-driven approaches are more viable than ever to provide new insights in cancer biology.

Computational tools are the bridge between these data and insight. Algorithms are designed to answer questions that are either experimentally too laborious or even infeasible. Consequentially, we have tools to nominate driver genes by analyzing patterns of genome-wide somatic mutations [2]; to discover molecular subtypes with distinct expression, copy number, and mutational features [3]; and to predict many fundamental characteristics of cancer samples, such as the average telomere length [4], purity [5], and even the abundance of infiltrating stromal and immune cells [6]. The power of such predictions lies in the amount of data, as associating these characteristics with cancer genomic and clinical parameters in thousands of cases permits the discovery of the subtlest links that otherwise would be buried deep in the data.

Thanks to a vibrant community, we usually have arrays of tools developed to address the same sets of questions. The availability of alternatives is critically important as no tool is guaranteed to grasp the full complexity of cancer genomic data. Take mutation calling, for example: more than a dozen mutation detection tools are available on the market [7]. The choice of which tool to use in a project often relies on popularity, ease of use, and demand for resources. While performance should be the best measure, it is more often that a tool is suggested to outperform competitors in benchmarking experiments conducted by its own developers. In light of this, community efforts such as the ICGC-TCGA DREAM challenges [8] are being established to provide a common benchmark reference to ensure fair and transparent comparisons.

For less widespread applications a consensus benchmark is likely not readily available. This does not suggest in any way that the object of the research is less significant. An example is deconvolution of bulk tumor expression. Pathologists have long learned that tumors are admixtures of malignant cells, fibroblasts, blood vessels, and immune cells. Systematic analysis demonstrates a continuum of tumor impurity, not only within a cancer type but also across cancer tissues of origin [5]. The tools discussed in two correspondence articles [9, 10], CIBERSORT and TIMER, developed by Newman et al. [11] and Li et al. [12], respectively, both aim to delineate admixture at the transcriptome level into immune cell types. While CIBERSORT takes on 22 cell types, TIMER focuses on 6, arguing that including too many variables would introduce statistical collinearity and lead to non-biological associations. Furthermore, Li et al. [12] assert that CIBERSORT was developed based on microarrays and is thus unsuitable for the analysis of RNA-seq data. In response, Newman and colleagues disputed these arguments and suggested that TIMER failed to normalize the immune cell estimates for total amount of leukocytes, which they believe could explain the observed discrepancies between the results generated by the two tools [9].

These correspondence articles clarify important details of both tools, which in our opinion are beneficial to end users. Furthermore, they invite discussions on what are the best strategies and common pitfalls in benchmarking computational tools. Here we summarize, in our view, useful sources for benchmarking experiments.

1. Ground truth. A dataset produced by gold standard experimental approaches is the strongest evidence for validation. To name a few: fluorescence in situ hybridization (FISH) for the validation of absolute copy number predictions, and the quantitative PCR-based telomeric repeat amplification protocol (TRAP) assay for the validation of telomerase activity prediction, etc. In some circumstances the property of interest is challenging to quantify and experiments can be designed to create an artificial reference. For example, a dilution series is able to provide an artificial sample with known proportions of mixtures, which can be compared to computational estimates such as purity. When using an experimental dataset as the benchmarking reference, it is important to keep in mind the context in which computational and experimental data align. As an exaggerated example, one would be ridiculed to validate a novel driver mutation found in breast cancer in a glioma cell line. In reality, however, we are tempted to make extrapolations based on limited sets of experiments. Acknowledging that we cannot practically test all possible scenarios, we stress that context should be carefully considered when applying or designing experimental data for benchmarking.
2. Simulation. Often times a gold standard is simply impossible to get, and this dilemma applies to many cancer genome analyses. For instance, we do not have a complete list of bona fide DNA structural rearrangements (SVs) or somatic mutations that allow us to evaluate the sensitivity and specificity of calling tools, albeit it is straightforward to validate the returned candidate events. In cases like this, we resort to other means, such as simulation. Generating a good simulation dataset takes deep understanding of the simulated object and addition of appropriate noise levels. Details are important as to how well the simulated data reflect the real case scenario. We encourage all authors to share their code on public platforms such as GitHub or SourceForge to enhance transparency and reproducibility regardless of requirements from publishers.
3. Literature and public resources. Literature represents a wealth of information. However, it is not uncommon to find contradictory evidence from the literature to either prove or disprove a conclusion. For this reason, citing one or a few references does not significantly strengthen benchmarking, in our view. Knowledge bases such as the Cancer Gene Census [13] are expert curations of publications and are more reliable sources. The same rationale might be extended to databases that collect data through text mining, which replaces human subjectivity with sematic parsing and machine learning. Benchmarking carries more weight as long as it is done with systematically collected data rather than hand-picked examples.
4. Other tools. Competing tools are usually compared with rather than benchmarked to. However, consensus results returned by multiple tools are presumably more reliable if they diverge in their premise and technical details. A consensus can be intuitively defined when two tools are enlisted but the complexity rises sharply upon inclusion of multiple tools, in which case careful evaluation is warranted to ensure proper stringency. In another setting, a new tool can be benchmarked to an existing tool addressing the same question but with different modalities of input data. This is particularly useful as such comparisons may provide insights beyond benchmarking.

Despite its importance, benchmarking is only a part of the tool development cycle. End users should be invited to the dialogue as they are the ones that apply and test tools in projects where they possess intimate knowledge that tool developers may not have. In this sense benchmarking of a tool occurs not only before but also after its publication.
